# *Ganoderma tsugae* Extract Inhibits Growth of HER2-Overexpressing Cancer Cells via Modulation of HER2/PI3K/Akt Signaling Pathway

**DOI:** 10.1155/2013/219472

**Published:** 2013-04-07

**Authors:** Han-Peng Kuo, Shih-Chung Hsu, Chien-Chih Ou, Jhy-Wei Li, Hsiu-Hsueh Tseng, Tzu-Chao Chuang, Jah-Yao Liu, Shih-Jung Chen, Muh-Hwan Su, Yung-Chi Cheng, Wei-Yuan Chou, Ming-Ching Kao

**Affiliations:** ^1^Department of Biological Science and Technology, College of Life Sciences, China Medical University, 91 Hsueh-Shih Road, Taichung 40402, Taiwan; ^2^Kang-Ning Junior College of Medical Care and Management, Taipei 11486, Taiwan; ^3^Oncology New Drug Division, SynCore Bio, Taipei 11070, Taiwan; ^4^Department of Pathology, Da-Chien General Hospital, Miaoli 36052, Taiwan; ^5^Graduate Institute of Life Sciences, National Defense Medical Center, Taipei 11490, Taiwan; ^6^Department of Chemistry, Tamkang University, Tamsui, New Taipei 25137, Taiwan; ^7^Department of Obstetrics & Gynecology, Tri-Service General Hospital, Taipei 11490, Taiwan; ^8^Luo-Gui-Ying Fungi Agriculture Farm, Taoyuan 33043, Taiwan; ^9^Sinphar Group Headquarter, Sinphar Group, Yilan 26944, Taiwan; ^10^Department of Pharmacology, Yale University School of Medicine, New Haven, CT 06520-8066, USA; ^11^Department of Biochemistry, National Defense Medical Center, Taipei 11490, Taiwan

## Abstract

*Ganoderma*, also known as Lingzhi or Reishi, has been used for medicinal purposes in Asian countries for centuries. It is a medicinal fungus with a variety of biological properties including immunomodulatory and antitumor activities. In this study, we investigated the molecular mechanisms by which *Ganoderma tsugae* (GT), one of the most common species of *Ganoderma*, inhibits the proliferation of HER2-overexpressing cancer cells. Here, we show that a quality assured extract of GT (GTE) inhibited the growth of HER2-overexpressing cancer cells *in vitro* and *in vivo* and enhanced the growth-inhibitory effect of antitumor drugs (e.g., taxol and cisplatin) in these cells. We also demonstrate that GTE induced cell cycle arrest by interfering with the HER2/PI3K/Akt signaling pathway. Furthermore, GTE curtailed the expression of the HER2 protein by modulating the transcriptional activity of the *HER2* gene and the stability/degradation of the HER2 protein. In conclusion, this study suggests that GTE may be a useful adjuvant therapeutic agent in the treatment of cancer cells that highly express HER2.

## 1. Introduction

Human epidermal growth factor receptor 2 (HER2) is a 185-kDa transmembrane receptor tyrosine kinase (RTK), belonging to the epidermal growth factor receptor (EGFR) family, which contains four homologous members: EGFR/HER1, HER2, HER3, and HER4. Ligand stimulation induces dimerization of the HER receptor (homo- or heterodimer), which leads to self-phosphorylation (except for HER3) on tyrosine residues localized to the C-terminal domain of HER receptors. Then, the phosphorylated HER receptors (activated form) activate a variety of downstream signaling pathways, such as the phosphatidylinositol-3-kinase (PI3K)/Akt and the Ras/mitogen-activated protein kinase (MAPK) pathways, which in turn promote cell proliferation, survival, and metastasis [1].

Aberrant upregulation of HER2 is found in approximately 25–30% of breast cancers [2] and in 6–50% of ovarian cancers [3]. Patients with HER2-positive cancer have a high risk for diminished effectiveness of cancer treatments, increased cancer metastasis, and poor clinical outcomes [4]. Therefore, inhibition of HER2 expression or its kinase activity may be an effective approach for the treatment of HER2-overexpressing cancers. In fact, a number of HER2-targeting agents, including monoclonal antibodies (e.g., trastuzumab) and small-molecule tyrosine kinase inhibitors (e.g., lapatinib), have been developed for the treatment of cancers with HER2-overexpression [1]. However, there is still a need for novel therapies to treat HER2-overexpressing cancers. For example, traditional Chinese medicine (TCM) and botanical products are currently considered to be safer and may be used as alternative therapeutic agents for treatment of cancers that overexpress HER2 [5, 6].

*Ganoderma* (also known as Lingzhi) has a long history of use in folk medicines in Asian countries. *Ganoderma lucidum* (GL) and *Ganoderma sinense* (GS), listed in *Chinese Pharmacopoeia* (2010 edition) [7, 8], are two of the most common species of *Ganoderma* and have been used for medicinal purposes in China for centuries. The biological activities of GL and GS, particularly their immunomodulatory and antitumor properties, have been well documented [9]. In addition, *Ganoderma tsugae* (GT), another well-cultivated species of *Ganoderma*, has been shown to have many biological and pharmacological properties, such as antiautoantibody formation [10], antifibrosis [11], antiinflammation [12], and antioxidation characteristics [13]. A number of reports show that GT has growth-inhibitory effects in a variety of human cancer cells, such as MDA-MB-231 and MCF-7 breast cancer cells [14], COLO 205 colorectal cancer cells [15], A431 epidermoid carcinoma cells [16], Hep3B hepatoma cells [17], and H23 and H23/0.3 lung adenocarcinoma cells [18]. Although GT has antitumor activity in many human cancer cells, the mechanisms that underlie its growth-inhibitory effect on HER2-overexpressing cancer cells remain unclear.

In this study, we produced a quality assured extract of GT (GTE) and characterized its antitumor effects and relevant molecular mechanisms in HER2-overexpressing cancer cells *in vitro* and *in vivo*. Our results show that GTE inhibits cancer cell growth and induces cell cycle arrest via modulation of the HER2/PI3K/Akt signaling pathway. We also show that combining GTE with taxol or cisplatin significantly slows the growth of HER2-overexpressing cancer cells, indicating a potential use of GTE in the treatment of cancers that overexpress HER2.

## 2. Materials and Methods

### 2.1. Cell Culture

Human ovarian carcinoma cell lines, SKOV-3 (HER2^high^) and OVCAR-3 (HER2^low^), and breast carcinoma cell lines, SKBR-3 (HER2^high^) and BT-474 (HER2^high^), were obtained from the American Type Culture Collection (ATCC, Manassas, VA, USA). The MCF-7/HER2 (HER2^high^) human breast carcinoma cell line (MCF-7 of an HER2-transfected stable line) was kindly provided by Dr. M. C. Hung (Department of Molecular and Cellular Oncology, University of Texas, M. D. Anderson Cancer Center, Houston, TX, USA). The MDA-MB-435/HER2 (HER2^high^) human melanoma cell line (MDA-MB-435 of an HER2-transfected stable line) was kindly provided by Dr. T. D. Way (Department of Biological Science and Technology, China Medical University, Taichung, Taiwan). All cells were cultured in DMEM/F12 medium (Gibco BRL, Grand Island, NY, USA) supplemented with 10% fetal bovine serum (FBS) in a humidified atmosphere of 5% CO_2_ at 37°C.

### 2.2. Chemicals and Antibodies

The thiazolyl blue tetrazolium bromide (MTT), cycloheximide (CHX), and N-acetyl-L-leucinyl-L-leucinyl-norleucinal (LLnL) were obtained from Sigma-Aldrich (St. Louis, MO, USA). Antibodies against cyclins D1 and E, p21, p27, phospho-Akt (Ser308), Akt1, and ubiquitin (Ub) were purchased from Santa Cruz Biotechnology, Inc. (Santa Cruz, CA, USA). Antibodies against phospho-PI3K, PI3K, phospho-Erk 1/2, and Erk 1/2 were purchased from Cell Signaling Technology, Inc. (Beverly, MA, USA). Antibodies against phospho-HER2 (Ab-18), HER2 (Ab-3), *β*-actin, and Ki-67 (Clone MIB-1) were purchased from Neomarkers Inc. (Fremont, CA, USA), Calbiochem (San Diego, CA, USA), Chemicon International Inc. (Temecula, CA, USA), and Dakocytomation Inc. (Carpinteria, CA, USA), respectively. Taxol (paclitaxel) was purchased from Bristol-Myers Squibb (Wallingford, CT, USA), and cisplatin was purchased from Pharmacia & Upjohn S.p.A. (Via Robert Koch 1.2, Milan, Italy).

### 2.3. Preparation of *Ganoderma tsugae* Extracts

*Ganoderma tsugae* (GT) was kindly provided by the Luo-Gui-Ying Fungi Agriculture Farm (with a registered name of Tien-Shen Lingzhi), Taoyuan, Taiwan. The extract of GT (GTE) was prepared as described previously [15]. Briefly, the powder of the GT fruiting body (5 g) was soaked in 99.9% methanol (200 mL), mixed, and shaken for 24 h on a rotating shaker. After centrifugation, the supernatant was poured through filter paper (Whatman, cat. no. 1001-110), and the residues were extracted with methanol two additional times as mentioned above. The filtrates were collected together and subjected to concentration under reduced pressure (i.e., evaporated to dryness under reduced pressure) to produce a brown gel-like GT extract (GTE). The yield was approximately 30%. The GTE was then prepared as a stock solution with methanol solvent (100 mg/mL) and stored at −80°C until use. For animal experiments, the dry GTE was redissolved in ethanol and diluted with a suspension solution (74.5% corn oil, 16% PEG-400, 4% Tween-80, 4% Cremophor EL, and 1.5% Ethanol, v/v) to a concentration of 10 mg/mL.

### 2.4. Quality Control of GTEs via Bioresponse Fingerprinting

The quality of the GTEs was assessed as described previously [18, 19]. Briefly, the genomic bioresponse to the GTEs was determined in SKOV-3 cells treated with 0.5 mg/mL of GTE. The total RNA was extracted from the GTE-treated cells, cleaned with a commercial kit (Qiagene RNA extraction kit, cat. no. 75144), and then used to obtain transcription profiles in GeneChip hybridization studies using Affymetrix technology. The changes in the individual gene expression levels obtained by the GeneChip experiments were measured by Affymetrix MAS 5.0 software. A statistical pattern comparison method from the PhytomicsQC platform, Phytomics Similarity Index (PSI), was applied to determine the batch-to-batch similarity of the botanical products. In general, clinically similar batches have a PSI more than 0.95.

### 2.5. Cell Proliferation Assay

Cell viability was determined using an MTT assay as previously described [6]. Briefly, cells were seeded at a density of 6,000 cells/well into 96-well plates and incubated overnight in a medium containing 10% FBS. After the cells adhered to the plate, various doses of GTE were added to the cells, and then the cultures were incubated at 37°C for 72 h. After incubation with MTT reagent (0.5 mg/mL) for 4 h, the relative viable cell numbers were directly proportional to the production of formazan crystals solubilized by DMSO. The final solution was measured using a spectrophotometer at a wavelength of 545 nm against a reference wavelength of 690 nm.

### 2.6. Soft Agar Colony Formation Assay

The effect of GTE on the potential for anchorage-independent growth was determined by soft agar colony formation assay as described previously [20] with slight modifications. The cells (2 × 10^4^ cells/well) were seeded in 6-well plates containing 0.7% base agar, 0.35% top agar and exposed to different concentrations of GTE or an equal volume of DMEM/F12 twice/week, and incubated at 37°C for 3 weeks. Colonies were stained with MTT reagent (5 mg/mL) and then photographed using a phase contrast microscope (100X) equipped with a CCD camera.

### 2.7. Flow Cytometric Analysis

For the analysis of the cell cycle, the phase distribution was detected by flow cytometry as described previously [6]. In brief, cells were incubated with GTE or the vehicle for 24 h and then fixed with ice-cold 70% ethanol overnight at 4°C. Prior to analysis, the cells were washed twice with PBS buffer and then incubated with propidium iodide (PI) solution (50 *μ*g/mL PI in PBS with 1% Tween-20 and 10 *μ*g RNase) for approximately 30 min in the dark at room temperature. The DNA content was measured using flow cytometry (BD FACS Canto). The FCS Express v2.0 software was used to analyze the results from the flow cytometric experiment.

### 2.8. Reporter Gene Assay

Cells were cotransfected with pHER2-luc (a HER2 promoter-driven luciferase gene plasmid construct) and pCMV-*β*-gal plasmids for 6 h and then incubated with GTE or the vehicle for 24 h. The HER2 promoter and *β*-galactosidase (*β*-gal) gene activity assays were performed as previously described [21]. The relative light units of luciferase activity were normalized to *β*-gal activity.

### 2.9. Semiquantitative Reverse Transcriptase-Polymerase Chain Reaction (RT-PCR)

Total RNA was isolated using TRIzol solution (Invitrogen, San Diego, CA, USA). Two micrograms of total RNA were used for first-strand cDNA synthesis. The appropriate primers (HER2 sense: 5′-CAATGGAGACCCGCTGAAC-3′; HER2 antisense: 5′-CAGTGCGCGTCAGGCTCT-3′; glyceraldehyde-3-phosphate dehydrogenase (GAPDH) sense: 5′-ACCACAGTCCATGCCATCAC-3′; GAPDH antisense: 5′-TCCACCACCCTGTTGCTGTA-3′) were used to perform the polymerase chain reaction (for 1 cycle at 94°C for 5 min, 32 cycles of 94°C for 15 s, 56°C for 30 s, and 72°C for 1 min with a final extension at 72°C for 5 min). The PCR products were separated by electrophoresis on a 1.2% agarose gel and detected by ethidium bromide (EtBr) staining.

### 2.10. Immunoprecipitation and Western Blotting

Proteins were extracted from the cells by the addition of lysis buffer (20 mM Hepes buffer pH 7.0, 10 mM KCl, 2 mM MgCl_2_, 0.5% NP-40, and protease inhibitors). Following cell lysis, the extracts were centrifuged at 16,000 ×g for 10 min at 4°C. The protein content of the supernatant was measured using the Bio-Rad protein assay kit. Immunoprecipitation was carried out as previously described [22] with a slight modification. Briefly, 300 *μ*g of total protein was incubated with anti-HER2 antibody overnight at 4°C, followed by protein A/G PLUS-Agarose (Santa Cruz) for 3 h at 4°C. The precipitates were resolved using sodium dodecyl sulfate polyacrylamide gel electrophoresis (SDS-PAGE) and then transferred onto a polyvinylidene fluoride (PVDF) membrane. For Western blotting as described previously [22], total protein (40 *μ*g) was loaded to the gel and blotted onto the PVDF membrane. The membranes were blocked using 5% nonfat milk in tris-buffered saline with Tween-20 (TBST) for 1 h at room temperature. After blocking, the PVDF membranes were incubated with primary antibodies for 1 h at room temperature, followed by an HRP-conjugated secondary antibody. The reactive signals were visualized using the Enhanced Chemiluminescence Kit (Amersham Biosciences, Arlington Heights, IL, USA). The bands were scanned and quantified using the ImageJ software.

### 2.11. Animal Experiments

The animal experiments were performed as described previously [15] with slight modifications. Briefly, 5 × 10^6^ SKOV-3 cells were subcutaneously implanted into the flank region of female BALB/c nude mice (BALB/cAnN.Cg-*Foxn1* ^*nu*^/CrlNarl). In total, 19 mice were used for this experiment; the tumor-implanted mice were treated with GTE (*n* = 12) or with the vehicle (*n* = 7), respectively. The GTE-treated mice were fed with GTE daily at a dose of 200 mg/kg (*n* = 5) or 1,000 mg/kg (*n* = 7) body weight; this dosing schedule was initiated when the developing tumor was approximately 50–100 mm^3^ in volume (approximately 2-3 weeks after the cancer cells were implanted). The tumor volume and body weight were monitored daily. The mice were sacrificed for pathology examinations when the tumor volume exceeded 1,000 mm^3^. The tumors were then completely excised from the subcutaneous tissue and weighed. Biochemical and hematological parameters were used to evaluate potential drug toxicity.

### 2.12. Immunohistochemical (IHC) Staining

SKOV-3 xenografted tumors and the surrounding tissues were excised, fixed in formalin, embedded in paraffin, cut in 4-*μ*m serial sections, and then placed onto glass slides. The tumor tissue-coated slides were then dewaxed with xylene and gradually hydrated with graded alcohols. After antigen retrieval was achieved by pressure-cooking in 10 mM citrate buffer (pH 6.0) for 6 min, immunostaining for Ki-67, HER2, and cyclin D1 (1 : 150 dilution) was then performed as described previously [23].

### 2.13. Statistical Analysis

All data are presented as the mean ± SD from three independent experiments. Statistical analysis was performed by one-way ANOVA. Differences between treatment groups were analyzed for significance by multiple comparisons using analysis of variance. *P < 0.05 and **P < 0.01 versus the vehicle-treated control group.

## 3. Results

### 3.1. Quality Control of GTE Using Bioresponse Fingerprint Analysis

The quality of TCMs are potentially influenced by many factors, such as the growth conditions and processing procedures [24]. To assess the quality of the GTE, the bioresponse fingerprints were analyzed by the pattern comparison method from the PhytomicsQC platform [19], which showed highly concordant biological profiles for GTEs (GTE1, GTE2, and GTE3), and extracted from three batches of GT, acting on SKOV-3 cells with a PSI value more than 0.95 (See Supplementary Figure S1A available online at http://dx.doi.org/10.1155/2013/219472). Under this PSI value, 376 genes with specifically altered expression (149 upregulations and 227 downregulations) were observed as bioresponse fingerprints of GTEs (See Supplementary Figure S1B). These results suggest that the GT powder products used in this study were stable, consistent, and of high quality.

### 3.2. GTE Inhibits Proliferation of HER2-Overexpressing Cancer Cells

**Figure 1:**
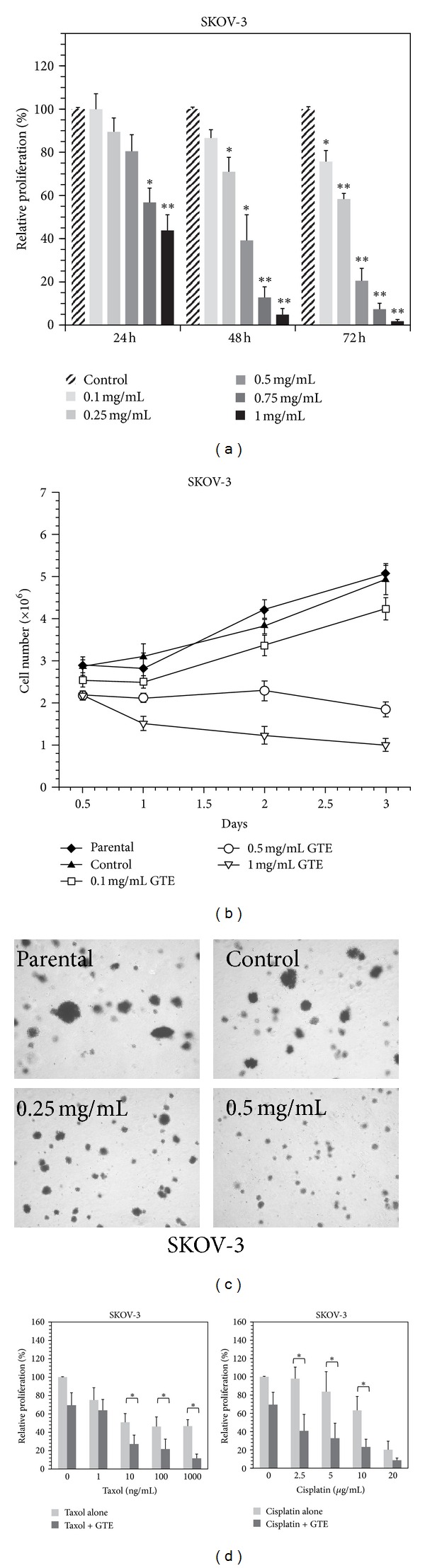
Effect of GTE on cell proliferation in HER2-overexpressing cancer cells. (a) SKOV-3 cells were treated with 0.5% methanol (vehicle control) or various concentrations of GTE (0.1, 0.25, 0.5, 0.75, and 1 mg/mL) for 72 h. Cell proliferation was measured using the MTT assay as described in Section 2. (b) SKOV-3 cells were treated with either vehicle control or GTE (0.1, 0.5, or 1.0 mg/mL) for 0.5, 1, 2, and 3 days. Cell numbers were determined using trypan blue staining. The parental SKOV-3 cells were not treated with vehicle (0.5% methanol) or GTE. (c) SKOV-3 cells were treated with different doses of GTE (0.25 and 0.5 mg/mL) twice a week for 3 weeks in the soft agar colony formation assay as described in Section 2. (d) SKOV-3 cells were treated with various concentrations of taxol (1, 10, 100, and 1000 ng/mL) or cisplatin (2.5, 5, 10, and 20 *μ*g/mL) with or without GTE (0.25 mg/mL) for 72 h. Cell proliferation was determined by MTT assay. The results are expressed as the mean ± SD of three independent experiments. *P < 0.05; **P < 0.01.

To determine whether GTE inhibits the growth of HER2-overexpressing cancer cells, we first evaluated the impact of GTE on cell proliferation using the MTT assay. As shown in Figure 1(a), the treatment of SKOV-3 cells (HER2^high^) with various concentrations of GTE (0.1–1 mg/mL) for 24–72 h resulted in significant dose- and time-dependent suppressive effects on the proliferation of SKOV-3 cells, accounting for a 0–56% reduction at 24 h, a 13–95% reduction at 48 h, and a 24–98% reduction at 72 h. Moreover, the trypan blue exclusion assay also clearly demonstrated that the GTE exhibited growth suppression effect at doses of 0.1–0.5 mg/mL while a less cytotoxic effect at 1.0 mg/mL on SKOV-3 cells (Figure 1(b)). Similar antiproliferative effects of GTE were also observed in other HER2-overexpressing cancer cells, for example, BT-474 and SKBR-3 (Supplementary Figures S2A and S2B). In addition, we assessed the influence of GTE on the potential for anchorage-independent growth, a hallmark of malignant cancer cells, using the soft agar colony formation assay. We found that GTE dramatically reduced anchorage-independent growth of SKOV-3 cells in a dose-dependent manner (Figure 1(c)). These results suggest that GTE is capable of inhibiting the proliferation of HER2-overexpressing cancer cells.

Resistance to chemotherapeutic agents (such as taxol and cisplatin) is a major problem in the treatment of cancers that overexpress HER2 [25, 26]. We therefore examined whether GTE could enhance the growth-inhibitory effects of anticancer drugs on SKOV-3 cells, by incubating the cells with both anticancer agents and GTE. As shown in Figure 1(d), GTE significantly enhanced the growth-inhibitory effects of taxol and cisplatin on SKOV-3 cells. We found that the proliferation of SKOV-3 cells was reduced by 30%, 45%, and 37% in cells exposed to GTE (0.25 mg/mL), taxol (10 ng/mL), and cisplatin (10 *μ*g/mL) alone, respectively. However, the proliferation of SKOV-3 cells was reduced by 73% and 77% in cells exposed to GTE combined with taxol and cisplatin, respectively. Similarly, we also found that GTE could increase the chemotherapeutic efficacy of anticancer drugs against other HER2-overexpressing cancer cell lines, for example, MDA-MB-453/HER2 (Supplementary Figures S3A and S3B). These findings suggest that GTE can chemosensitize HER2-overexpressing cancer cells to anticancer drugs (e.g., taxol and cisplatin).

### 3.3. GTE Induces G1 Phase Arrest by Modulating the Expression of Cell Cycle Regulatory Proteins

**Figure 2:**
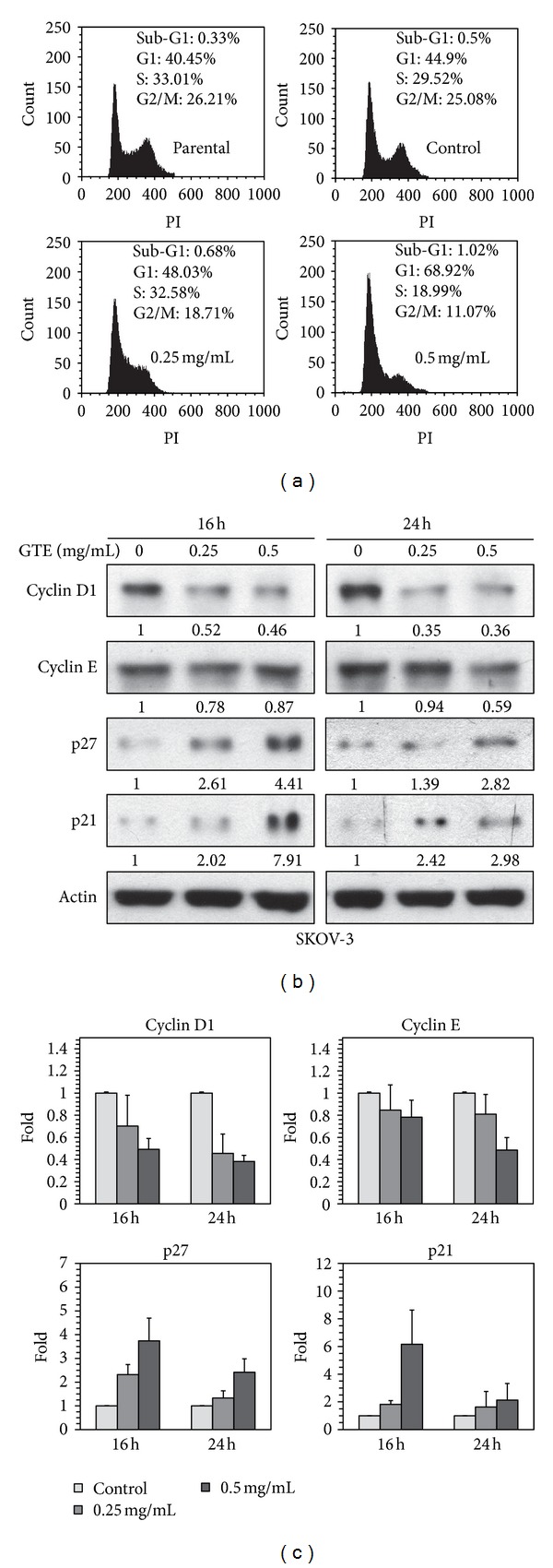
Effect of GTE on cell cycle distribution in HER2-overexpressing cancer cells. (a) SKOV-3 cells were treated with vehicle control (0.5% methanol) or various concentrations of GTE (0, 0.25, and 0.5 mg/mL) for 24 h. Cell cycle distribution was analyzed by flow cytometry as described in Section 2. The parental SKOV-3 cells were not treated with vehicle (0.5% methanol) or GTE. (b) SKOV-3 cells were treated with various concentrations of GTE (0, 0.25, and 0.5 mg/mL) for 16 h and 24 h. The expression of G1 phase regulators was determined by Western blotting as described in Section 2. (c) A histogram showing the relative protein levels from (b). Data are presented as the mean ± SD of three independent experiments. *P < 0.05 and **P < 0.01 versus the vehicle-treated control group.

As mentioned above, we observed a growth-inhibitory influence of GTE on SKOV-3 cells (Figures 1(a)–1(c)). To determine if the antiproliferative property of GTE was due to the disruption of cell cycle, flow cytometry was used to analyze the cell cycle change in SKOV-3 cells. As illustrated in Figure 2(a), treatment of SKOV-3 cells with GTE resulted in a distinct increase (approximately 24%) in the number of G1 phase cells at a concentration of 0.5 mg/mL GTE. This increase in the number of cells in the G1 phase was accompanied by a concordant decrease in the number of cells in the S and G2/M phases. Similar GTE-mediated cell cycle distribution patterns were observed in BT-474 (HER2^high^) cells (Supplementary Figure S4A). These findings suggest that GTE inhibits the growth of HER2-overexpressing cancer cells by modulating the progression of the cell cycle.

Different cell cycle regulators, such as cyclins, cyclin-dependent kinases (CDKs), and CDK inhibitors (CKIs), are involved in multiple cellular pathways that tightly regulate the progression of the cell cycle [27]. To elucidate the molecular mechanisms of GTE-induced cell cycle arrest, we assessed the impact of GTE on the expression of cell cycle regulators. We demonstrated that, after GTE treatment, the protein levels of cyclins D1 and E were downregulated, while the protein levels of p21 and p27 were upregulated in SKOV-3 cells (Figures 2(b) and 2(c)). Similarly, GTE also dramatically affected the expression of cell cycle regulators (e.g., cyclins D1 and E) in two more HER2-overexpressing cancer cell lines, that is, BT-474 (Supplementary Figure S4B) and SKBR-3 cells (data not shown). These results suggest that GTE inhibits cell growth by regulating the expression of cell cycle regulators in HER2-overexpressing cancer cells.

### 3.4. GTE Inhibits HER2/PI3K/Akt Signaling Cascades

**Figure 3:**
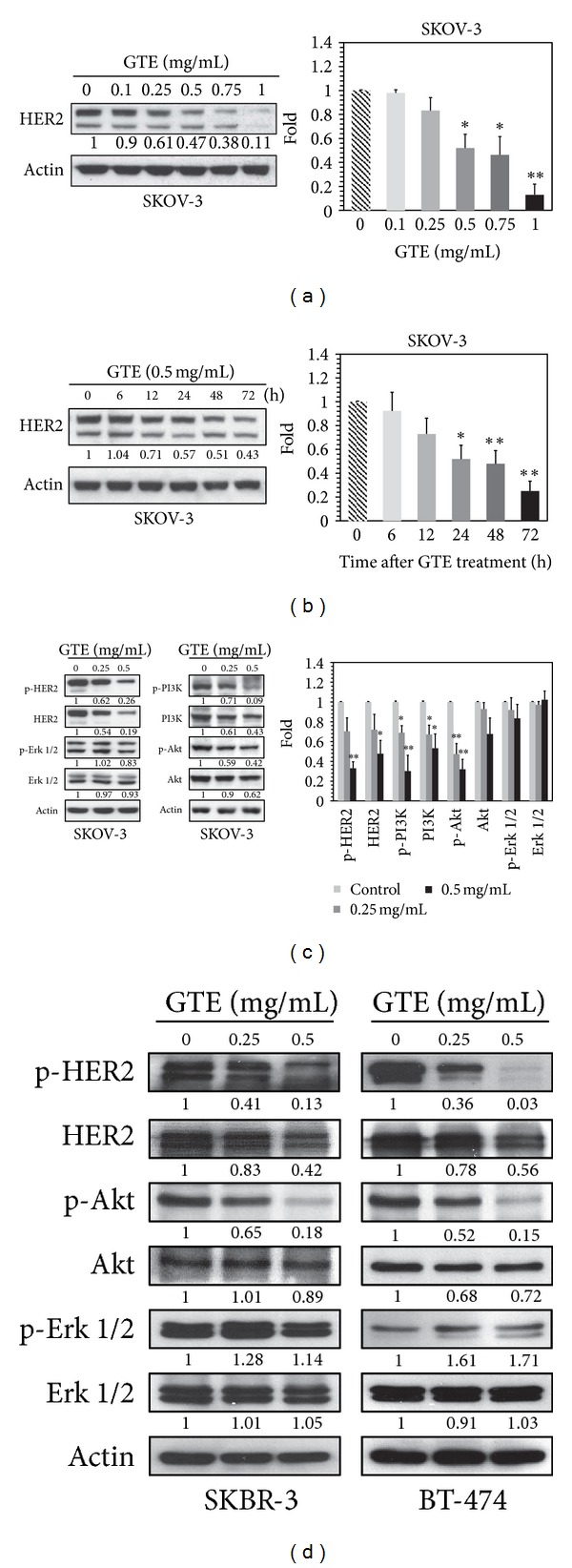
Effect of GTE on HER2/PI3K/Akt and Ras/MAPK signaling cascades in HER2-overexpressing cancer cells. (a) SKOV-3 cells were treated with various concentrations of GTE (0, 0.1, 0.25, 0.5, 0.75, and 1 mg/mL) for 24 h. The expression of HER2 protein was measured by Western blotting. (b) SKOV-3 cells were treated with 0.5 mg/mL GTE for 6, 12, 24, 48, and 72 h. The protein level of HER2 was determined by Western blotting. (c) Treatment of SKOV-3 cells with GTE (0, 0.25, or 0.5 mg/mL) for 24 h inhibited HER2/PI3K/Akt but not HER2/Erk signaling. (d) Treatment of SKBR-3 and BT-474 cells with GTE (0, 0.25, 0.5 mg/mL) for 24 h led to inhibition of the HER2/Akt but not the HER2/Erk signaling pathway. The results are expressed as the mean ± SD of three independent experiments. *P < 0.05; **P < 0.01.

Based on the results mentioned above, there was a significant growth-inhibitory effect of GTE on HER2-overexpressing cancer cells (Figure 1). We next explored whether the inhibition of proliferation was caused by regulating the expression of HER2 protein. As shown in Figures 3(a) and 3(b), treatment of SKOV-3 cells with GTE resulted in a marked dose- and time-dependent decrease in HER2 protein levels. Similarly, GTE also decreased the protein expression of HER2 in other HER2^high^ cell lines, such as SKBR-3, BT-474, and MCF-7/HER2 (Figure 3(d), Supplementary Figure S5A) and an HER2^low^ cell line, OVCAR-3 (Supplementary Figure S5B).

The HER2 signaling pathway is known to be associated with cell proliferation; therefore, we tested the impact of GTE on two main downstream pathways of HER2: the PI3K/Akt and Ras/MAPK signaling cascades [1]. As shown in Figure 3(c), GTE exhibited inhibitory effects on phospho-HER2, phospho-PI3K, and phospho-Akt without a noticeable reduction in phospho-Erk 1/2 in SKOV-3 cells. Moreover, GTE showed similar effects on phospho-HER2 and phospho-Akt in other HER2-overexpressing cell lines, for example, SKBR-3 and BT-474 (Figure 3d)). These data clearly indicate that GTE exerts inhibitory effects on the HER2/PI3K/Akt signaling cascades in cancer cells with HER2-overexpression.

### 3.5. GTE Downregulates HER2 Protein Expression by Modulating the Gene Expression and Protein Stability of HER2

**Figure 4:**
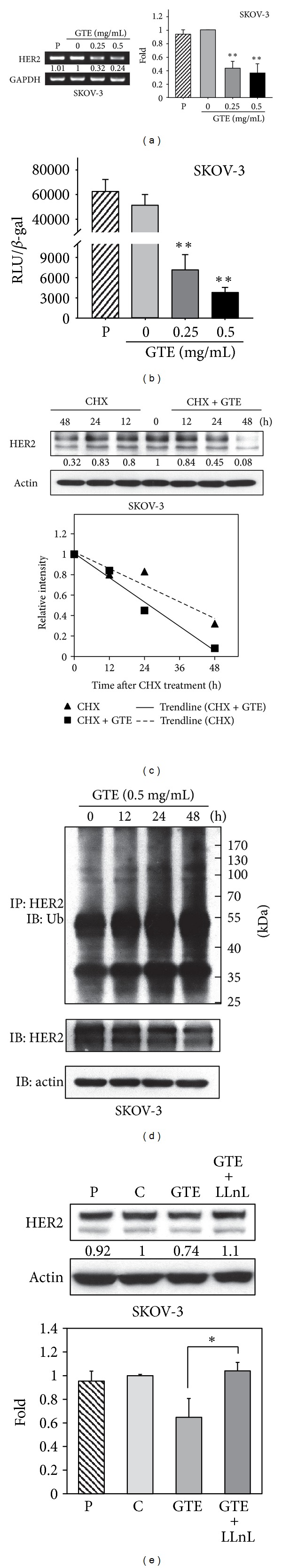
Effect of GTE on the gene expression and protein stability of HER2. (a) SKOV-3 cells were treated with GTE (0.25 or 0.5 mg/mL) or the vehicle for 24 h. The mRNA level of HER2 was measured by semiquantitative RT-PCR as described in Section 2. (b) SKOV-3 cells were transfected with a luciferase gene plasmid construct driven by HER2 promoter (pHER2-luc) for 6 h and then treated with various concentrations of GTE (0, 0.25, and 0.5 mg/mL) for 24 h. The activity of HER2 promoter was measured by a reporter gene assay, as described in Section 2. The relative light units (RLU) of luciferase activity were normalized against *β*-gal activity. (c) SKOV-3 cells were pretreated with 20 *μ*g/mL of cycloheximide (CHX) for 30 min and then treated with GTE (0.5 mg/mL) or the vehicle for 12, 24, and 48 h. Stability of HER2 was determined by measuring the protein's half-life. (d) SKOV-3 cells were treated with GTE (0.5 mg/mL) for 12, 24, and 48 h. To detect polyubiquitinated HER2 (HER2-Ub_(n)_), HER2 was immunoprecipitated and subjected to Western blot analysis using an antibody to ubiquitin. The total protein levels of HER2 and actin in the whole-cell extracts were also detected by Western blotting. (e) SKOV-3 cells were pretreated with proteasome inhibitor (LLnL) or the vehicle for 30 min and then treated with GTE (0.5 mg/mL) for 24 h. The protein level of HER2 was measured by Western blotting. P, parental SKOV-3 cells; C, vehicle control. Data are presented as the mean ± SD of three independent experiments. *P < 0.05 and **P < 0.01 versus the vehicle-treated control group.

As mentioned above, our results showed a dramatic inhibitory influence of GTE on the expression of HER2 protein in HER2-overexpressing cancer cells (Figure 3). To determine the underlying molecular mechanisms of the GTE-mediated downregulation of HER2, we tested the effect of GTE on the transcriptional activity of *HER2* gene. The expression of HER2 mRNA was distinctly decreased in SKOV-3 (Figure 4(a)) and BT-474 (Supplementary Figure S6A) cells exposed to 0.25 and 0.5 mg/mL of GTE for 24 h, as determined by RT-PCR. Furthermore, the reporter gene assay indicated that GTE decreased the HER2 promoter activity in a dose-dependent manner in SKOV-3 cells (Figure 4(b)). Consistent with the decreased expression of HER2 protein, both the mRNA level and the promoter activity of HER2 were downregulated by GTE. Taken together, we conclude that GTE depletes the protein levels of HER2 via modulation of the *HER2* gene activity.

Because an overall decrease in protein stability could also be responsible for the reduced HER2 protein levels, we examined the effect of GTE on HER2 protein stability and found that the half-life of HER2 was clearly shortened by GTE treatment in SKOV-3 (Figure 4(c)) and BT-474 (Supplementary Figure S6B) cells. In general, proteins such as HER2 are tagged with polyubiquitin and then degraded by the ubiquitin-proteasome system (UPS). We tested whether the GTE-mediated HER2 protein stability was due to the activation of the UPS. As shown in Figure 4(d), the amount of polyubiquitinated HER2 (HER2-Ub_(n)_) protein was significantly increased in SKOV-3 cells exposed to 0.5 mg/mL GTE for 24 or 48 h. In addition, the treatment of SKOV-3 cells with LLnL, a proteasome inhibitor, effectively prevented the GTE-mediated degradation of HER2 protein (Figure 4(e)). These observations suggest that the curtailment of HER2 by GTE may also occur through the induction of HER2 protein instability/degradation.

### 3.6. GTE Inhibits the Growth of SKOV-3 Xenografted Tumors by Modulating HER2 Protein

**Figure 5:**
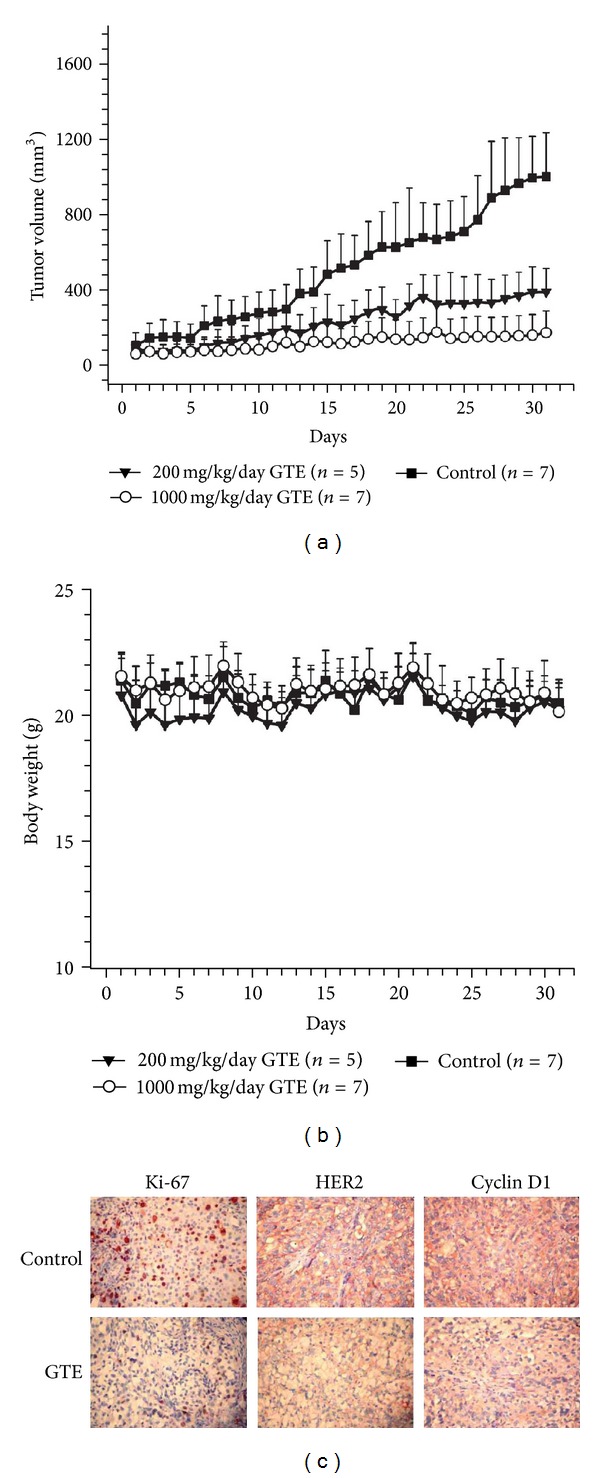
Effect of GTE on the growth of SKOV-3 xenografted tumors *in vivo*. (a) Tumor growth rate was significantly slower in the GTE-treated group (200 mg/kg/day, *n* = 5; or 1,000 mg/kg/day, *n* = 7) versus the control group (*n* = 7). The tumor volumes were estimated from the caliper measurements of three dimensions of the tumor. The estimated tumor volumes were calculated as *L* × *W* ^2^ × 0.5, where *L* is the major axis and *W* is tumor width. The results are represented as the mean ± SD. (b) The body weight of nude mice was not significantly different between the control and GTE-treated groups. (c) Downregulation of Ki-67, HER2, and cyclin D1 expression by GTE in SKOV-3 xenografted tumors on nude mice. The IHC analysis was performed on SKOV-3-induced xenografted tumors. The two representative specimens appear to show that GTE-treated mice (200 mg/kg/day) have lower protein expression than vehicle controls, for Ki-67, HER2, and cyclin D1 (400X magnification).

To determine the potential for anticancer effects of GTE* in vivo*, we used xenografted tumor-bearing nude mice. After the volume of the SKOV-3 xenografted tumors reached approximately 50–100 mm^3^, the mice were orally (p.o.) administered either GTE (200 and 1,000 mg/kg/day) or vehicle for 31 days. As illustrated in Figure 5(a), the nude mice treated with 200 or 1,000 mg/kg/day of GTE exhibited a marked inhibition in the growth of SKOV-3-implanted tumors relative to that of the control group. There was no significant alteration in the body weights of the nude mice with or without GTE treatment, indicating GTE had no apparent toxicity (Figure 5(b)). In addition, in comparison to the vehicle controls, the expression of Ki-67 protein, a proliferation marker, was significantly decreased in GTE-treated tumors (Figure 5(c)), indicating that GTE inhibited cell proliferation of SKOV-3 xenografted tumors *in vivo*.

In our *in vitro* studies, we showed that GTE inhibited cell proliferation and induced G1 cell cycle arrest in HER2-overexpressing cancer cells through the modulation of HER2 expression. To determine the underlying molecular mechanisms of the GTE-mediated anticancer effect observed in the SKOV-3 xenografted tumors, tumor sections were immunostained for HER2 protein and cyclin D1, the first cyclin that is activated during G1/S phase progression. In comparison to the control group, the staining intensities of HER2 and cyclin D1 were dramatically downregulated in GTE-treated tumor cells (200 mg/kg/day) (Figure 5(c)). Together, these data suggest that GTE inhibited tumor cell proliferation by inducing cell cycle arrest and modulating the HER2 pathway *in vitro* and *in vivo*.

## 4. Discussion

HER2-overexpression is associated with a high risk for cancer metastasis and a poor response to antitumor therapies [4]. Treatment with therapeutic agents that specifically target cancer cells with HER2-overexpression, such as lapatinib and trastuzumab, has improved clinical outcomes. In addition to the anticancer agents, a number of TCMs and botanical products have been shown to be effective and useful adjuvant agents for the treatment of HER2-overexpressing cancer [5, 6, 26]. *Ganoderma tsugae* (GT), one of the most common species of *Ganoderma* cultivated in Taiwan, has been shown to have antiproliferative effects on human cancer cells [15, 16, 18]. In this study, we report for the first time that the extract of GT (GTE) has a distinct growth-inhibitory effect on HER2-overexpressing cancer cells *in vitro* (Figures 1(a)–1(c)) and *in vivo* (Figure 5(a)).

Perturbation of cell cycle progression in cancer cells is a useful strategy to arrest cancer growth [28]. Furthermore, cell cycle arrest also provides an occasion for cells to undergo either repair or programmed cell death. A number of TCMs (e.g., GT) exhibit marked growth-inhibitory effects on cancer cells via disruption of cell cycle progression. Previous reports show that GT inhibits cell proliferation by inducing cell cycle arrest in the G2/M phase in Hep3B hepatoma and COLO205 colorectal cancer cells [15, 17] and in the S phase in H23/0.3 lung adenocarcinoma cells [18]. In this study, our *in vitro* results indicate that GTE treatment induces G1 phase arrest via modulation of cell cycle regulators (e.g., cyclins D1 and E, p21, and p27) in HER2-overexpressing SKOV-3 ovarian cancer and BT-474 breast cancer cells (Figure 2 and Supplementary Figure S4). The varying effects of GTE on the cell cycle may be due to cell-type specificity and/or result from modulation of different signal transductions and cell cycle regulatory molecules.

Two major therapeutic approaches to the treatment of HER2-overexpressing cancers involve agents that curtail the expression and activation/phosphorylation of the HER2 receptor [29]. In this study, we demonstrate that GTE downregulates both the level of HER2 and its phosphorylated form in SKOV-3, BT-474, and SKBR-3 cells (Figure 3). We surmised that the inhibitory effect of GTE on the levels of phospho-HER2 may be due to its inhibition of the expression of HER2. In agreement with this hypothesis, we observed a significant decrease in the expression of HER2 mRNA (Figure 4(a)) and the activity of its promoter (Figure 4(b)) following treatment with GTE. Moreover, we have established a number of HER2 promoter deletion constructs (F1: −1067~−103, F2: −871~−103, F3: −495~−103, and F4: −207~−103) and found that GTE interacts with the HER2 promoter in the −871~−495 region (unpublished data). Based on Genomatix software predictions, there are several putative transcription factor binding sites located in this area, such as T-cell factor (TCF), forkhead-box K2 (FOXK2), and GATA-binding protein 2 (GATA2). Therefore, further studies are needed to clarify the molecular basis by which the transcription of the *HER2* gene is regulated to ultimately aid in the development of better strategies for the treatment of cancers with HER2-overexpression.

We also investigated the regulation of HER2 protein stability/degradation as another possible explanation as to how GTE controls HER2 protein expression. We found that the half-life of the HER2 protein is noticeably reduced by GTE in SKOV-3 (Figure 4(c)) and BT-474 cells (Supplementary Figure S6B). This observation led us to hypothesize that the decreased stability of the HER2 protein may be due to the induction of polyubiquitination of HER2 by GTE (Figure 4(d)), leading to its degradation by the proteasome complex. We used LLnL, a proteasome inhibitor, to confirm that the effect of GTE on the degradation of HER2 protein involves the activation of the ubiquitin-proteasome system (Figure 4(e)). Furthermore, several molecules, such as heat shock protein 90 (Hsp90), casitas B-lineage lymphoma (c-Cbl), and peptidyl-prolyl cis/trans isomerase 1 (Pin1), are reported to be required for the maintenance of the stability and activation of HER2 [30–32]. It would be worthwhile to determine if these molecules are involved in the GTE-induced degradation/instability of the HER2 protein.

Generally, cancer cells overexpressing HER2 respond poorly to chemotherapeutic agents. Suppression of the HER2 pathway by HER2-targeting therapeutics potentiates the anticancer activity of chemotherapeutic agents in the treatment of HER2-overexpressing cancers [25, 33]. A number of reports show that the combined usage of some extracts from TCMs (e.g., coptis rhizome and glycyrrhizae radix) with antitumor agents results in synergistic growth inhibition in cancer cells [34, 35]. It has also been reported that combining anticancer agents with GTE slows the growth rate of cancer cells [15, 18]. Herein, we demonstrate for the first time that the combined usage of GTE with taxol (Figure 1(d)), cisplatin (Supplementary Figure S3), or doxorubicin (data not shown) results in synergistic growth inhibition of HER2-overexpressing cancer cells. These results indicate that GTE may be a promising adjuvant therapeutic agent in the treatment of cancers with HER2-overexpression.

**Figure 6:**
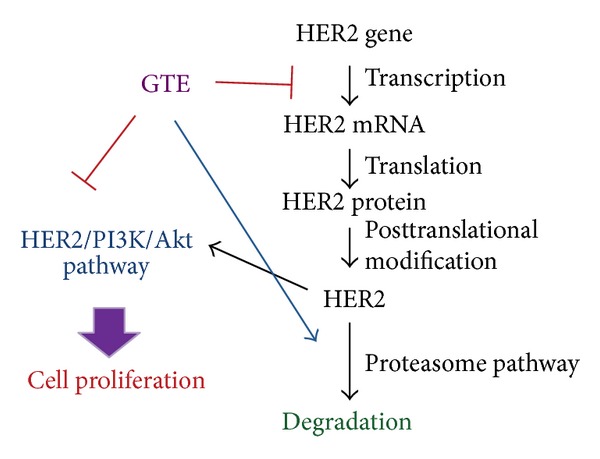
A schematic model of the GTE-mediated antiproliferative effect on HER2-overexpressing cancer cells. Ligand stimulation induces the activation of the HER2 receptor, which in turn activates the PI3K/Akt signaling pathway and then promotes cell growth and survival. After GTE treatment, the proliferation is inhibited because of an induction of cell cycle arrest. The GTE-mediated growth repression coincides with a reduction in the transcriptional activity of *HER2* gene and an induction in the degradation of HER2 protein, leading to a downregulation of the HER2/PI3K/Akt pathway.

In conclusion, we provide a schematic presentation of possible molecular mechanisms *in vitro* and *in vivo* for the inhibitory effects of GTE on the proliferation of HER2-overexpressing cancer cells (Figure 6). Our results indicate that GTE induces G1 cell cycle arrest via regulation of the HER2/PI3K/Akt signaling pathway, thereby leading to a reduction in the growth of cancer cells overexpressing HER2. Our data also demonstrate that the depletion of HER2 protein by GTE involves an inhibition in the transcriptional activity of the *HER2* gene and an increase in the proteasome-dependent degradation of the HER2 protein. In addition, we have also shown that a combination of GTE with anticancer drugs (e.g., taxol, cisplatin, and doxorubicin) exerts synergistic growth-inhibitory effect on HER2-overexpressing cancer cells. Taken together, our findings suggest that GTE may be a useful and effective adjuvant therapeutic agent for the treatment of cancers that highly express HER2.

## Supplementary Material

In order to provide further evidences for this study, the following experiments were performed and the results were demonstrated as supplementay material to relate to the relevant results shown in the main text. The supplementary material includes:
(1). Quality control of *Ganoderma tsugae* extracts (GTEs) was tested by the bioresponse fingerprinting technique (Ref. 18 & 19) (Fig. S1).
(2). GTE also affected the proliferation of HER2-overexpressing breast cancer BT474 and SKBR-3 cells (Fig. S2) (related to Figure 1a).
(3). GTE also sensitized the anticancer effects of taxol and cisplatin on HER2-overexpressing breast cancer MDA-MB-435/HER2 cells (Fig. S3) (related to Figure 1d).
(4). GTE also affect the cell cycle distribution of HER2-overexpressing breast cancer BT474 cells (Fig. S4) (related to Figure 2).
(5). GTE also affected the expression of HER2 protein in breast cancer MCF-7/HER2 (HER2*high*) and OVCAR-3 (HER2*low*) cells (Fig. S5) (related to Figures 3(a,b).
(6). GTE also affected the expression of HER2 mRNA and the stability of HER2 protein in breast cancer BT-474 cells (Fig. S6) (related to Figures 3(c,d).
