# Supplementary material for: Ganoderma tsugae Extract Inhibits Growth of HER2-Overexpressing Cancer Cells via Modulation of HER2/PI3K/Akt Signaling Pathway
Source: Evid Based Complement Alternat Med. 2013 Apr 7;2013:219472. doi: 10.1155/2013/219472 (PMC3638576; doi:10.1155/2013/219472)
Supplement: Supplementary file 1 — In order to provide further evidences for this study, the following experiments were performed and the results were demonstrated as supplementay material to relate to the relevant results shown in the main text. The supplementary material includes: (1). Quality control of Ganoderma tsugae extracts (GTEs) was tested by the bioresponse fingerprinting technique (Ref. 18 & 19) (Fig. S1). (2). GTE also affected the proliferation of HER2-overexpressing breast cancer BT474 and SKBR-3 cells (Fig. S2) (related to Figure 1a). (3). GTE also sensitized the anticancer effects of taxol and cisplatin on HER2-overexpressing breast cancer MDA-MB-435/HER2 cells (Fig. S3) (related to Figure 1d). (4). GTE also affect the cell cycle distribution of HER2-overexpressing breast cancer BT474 cells (Fig. S4) (related to Figure 2). (5). GTE also affected the expression of HER2 protein in breast cancer MCF-7/HER2 (HER2high) and OVCAR-3 (HER2low) cells (Fig. S5) (related to Figures 3(a,b). (6). GTE also affected the expression of HER2 mRNA and the stability of HER2 protein in breast cancer BT-474 cells (Fig. S6) (related to Figures 3(c,d). [file 219472.f1.pdf]

Supplementary Fig. S1

A

	GT1	GT2	GT3
GT1	1	0.9722	0.9558
GT2	-	1	0.9593
GT3	-	-	1

B

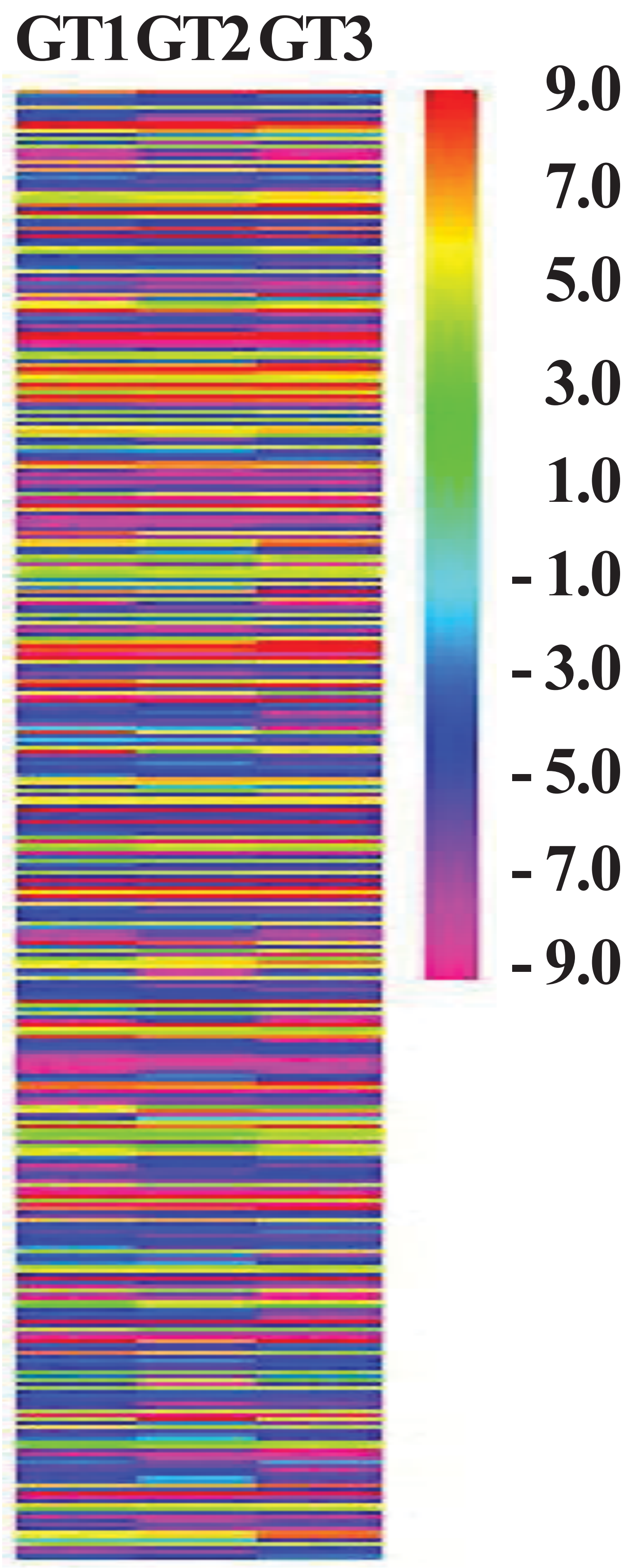

Supplementary Fig. S1: Quality control of *Ganoderma tsugae* extracts (GTEs). (A) Phytomics Similarity Index (PSI) (judged by PhytoViewerBR2.2, PhytoCeutica, Inc.). (B) The bioresponse fingerprint (BF) of GTEs. BF expressed as a color graph.

Supplementary Fig. S2

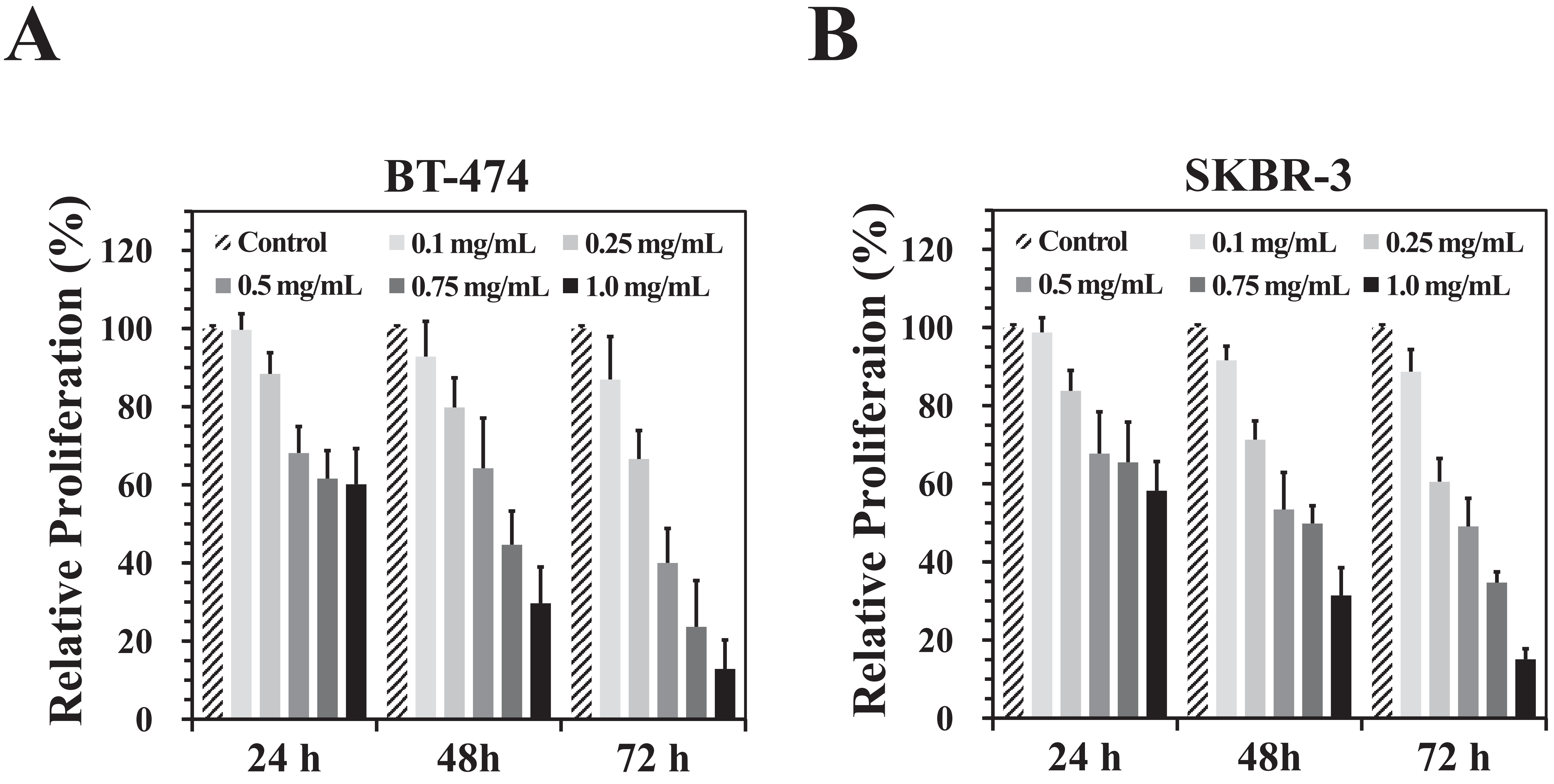

Supplementary Fig. S2: Effect of GTE on cell proliferation of HER2-overexpressing cancer cells. BT-474 (A) and SKBR-3 (B) cells were treated with various concentration of GTE (0.1, 0.25, 0.5, 0.75, and 1 mg/mL) for 72 h. Cell proliferation was determined using the MTT assay.

Supplementary Fig. S3

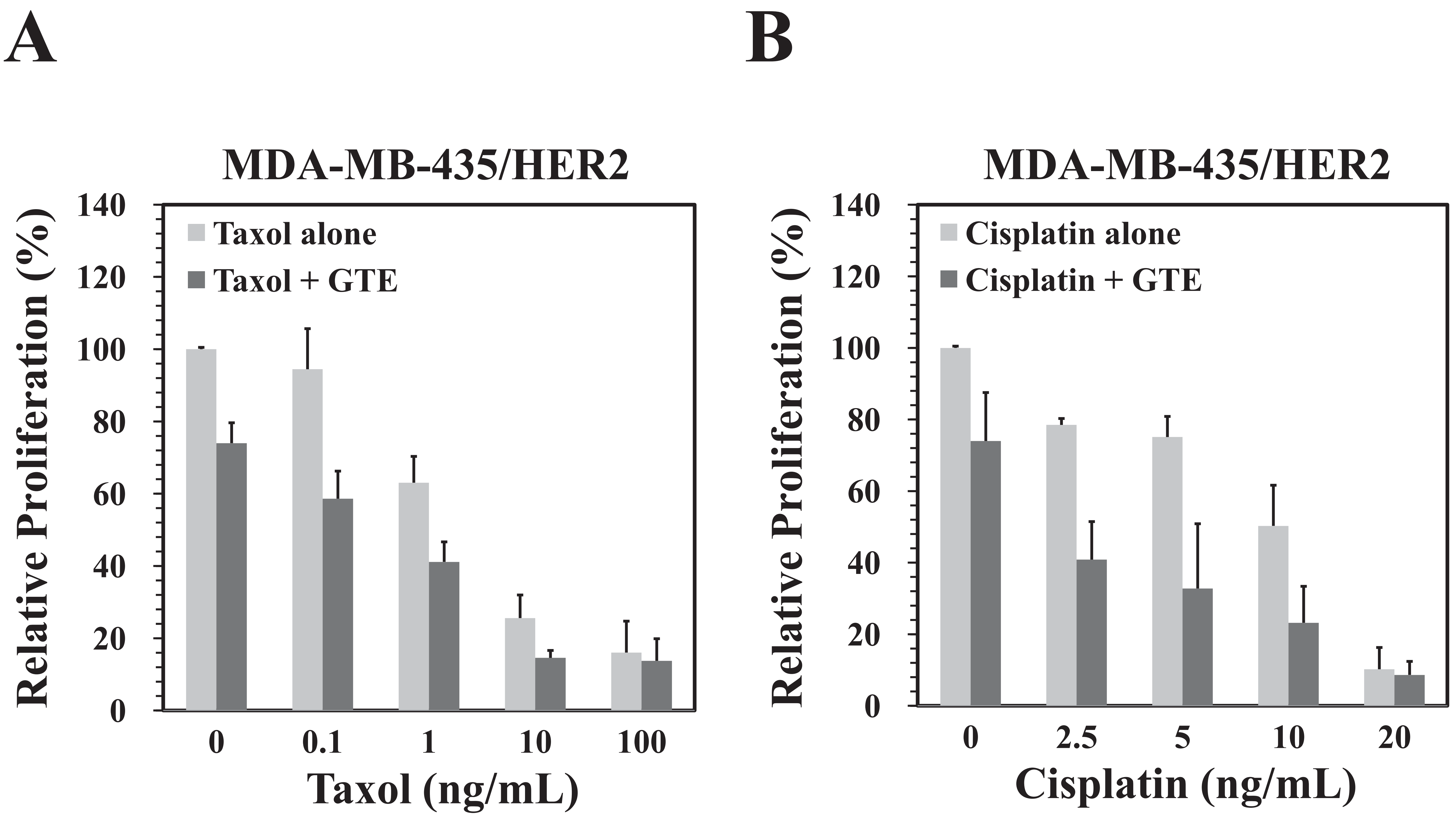

Supplementary Fig. S3: GTE sensitizes the anticancer effects taxol and cisplatin on HER2-overexpressing cancer cells. MDA-MB-435/HER2 cells were treated with various concentration of taxol (A) or cisplatin (B) with or without GTE (0.25 mg/mL) for 72 h. Cell proliferation was determined by MTT assay.

Supplementary Fig. S4

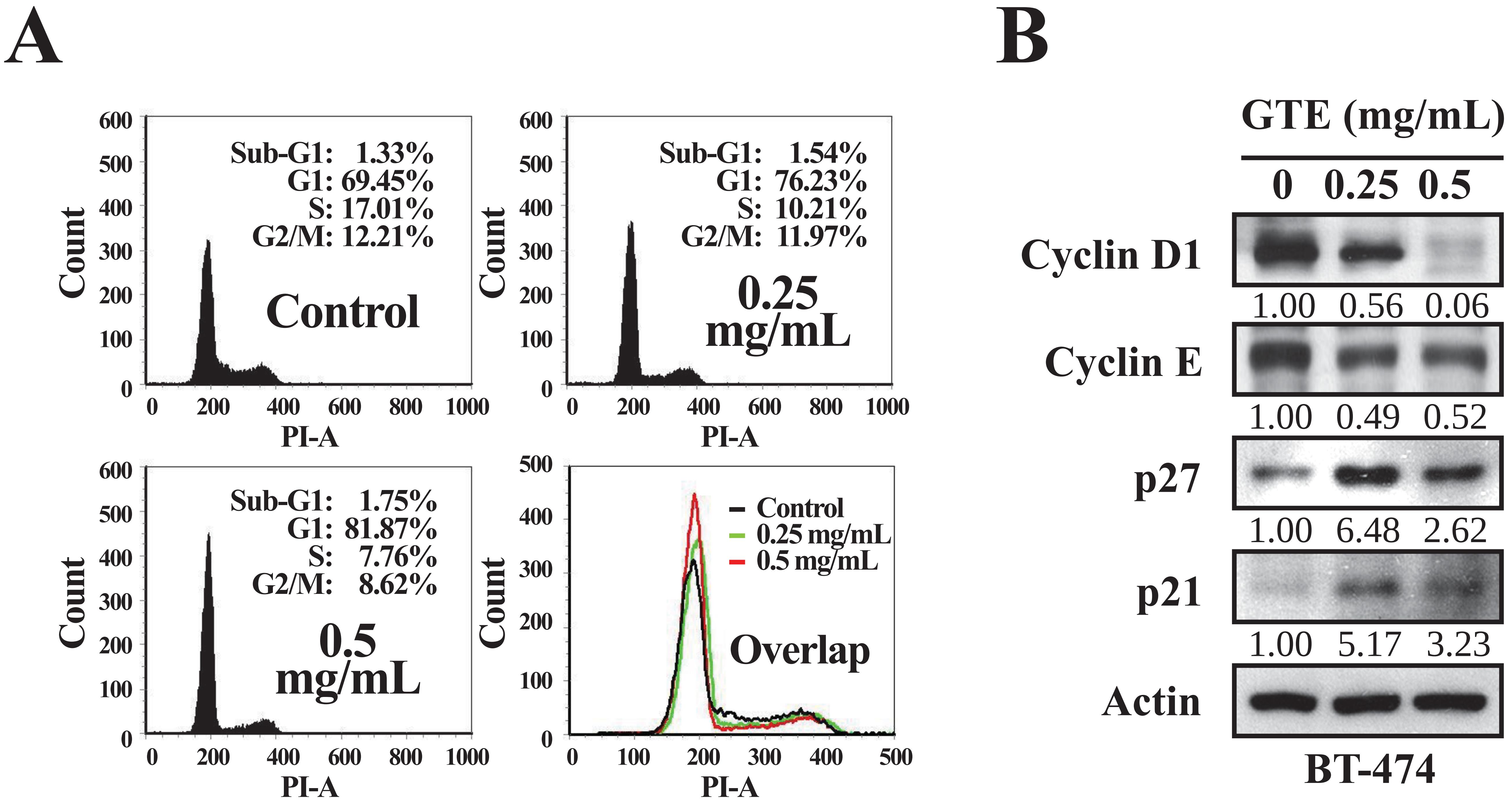

Supplementary Fig. S4: Effect of GTE on cell cycle of HER2-overexpressing cancer cells. BT-474 cells were treated with GTE (0.25 or 0.5 mg/mL) for 24 h. Distribution of cell cycle phases (A) and expression of cell cycle regulators (B) were analyzed by flow cytometry and Western blotting, respectively.

Supplementary Fig. S5

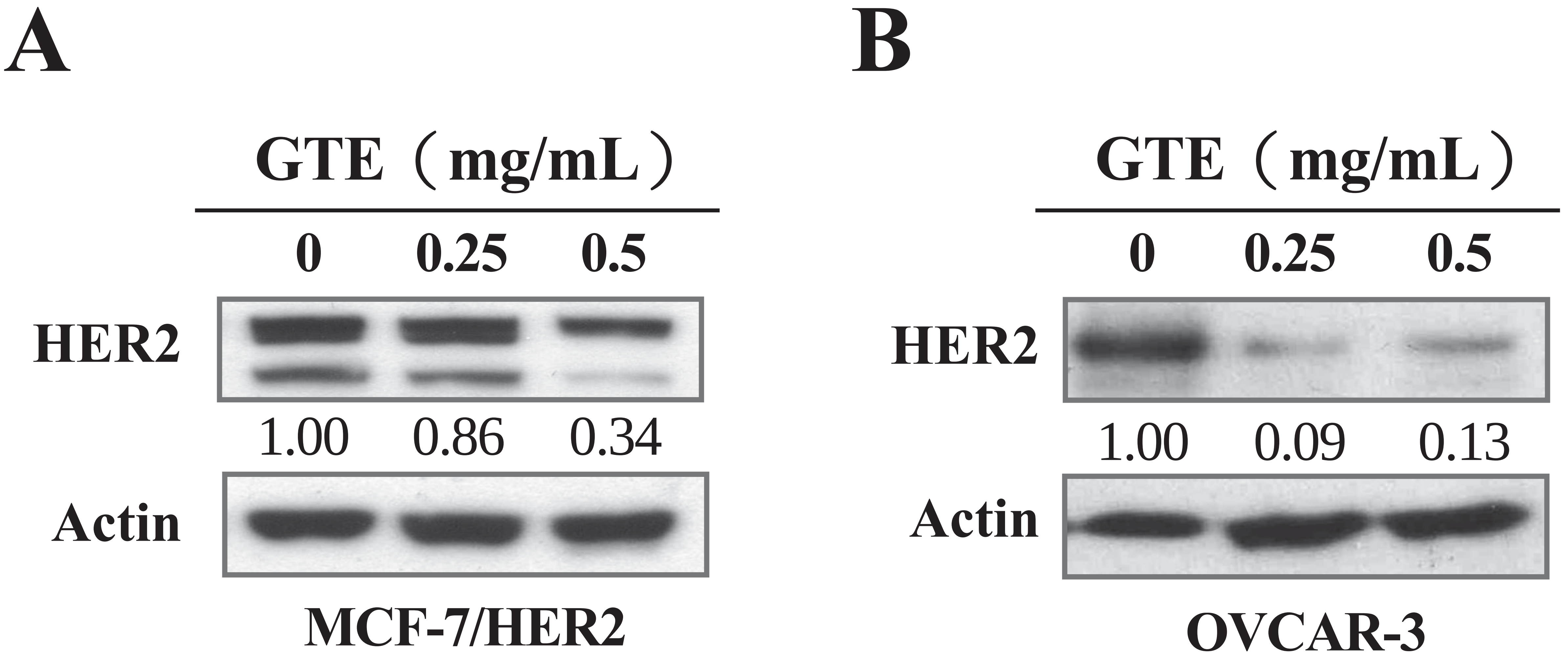

Supplementary Fig. S5: Effect of GTE on the expression of HER2 protein. MCF-7/HER2 ($HER2^{high}$) (A) and OVCAR-3 ($HER2^{low}$) (B) cells were treated with GTE (0.25 or 0.5 mg/mL) for 24 h. The protein expression of HER2 was measured by Western blotting.

Supplementary Fig. S6

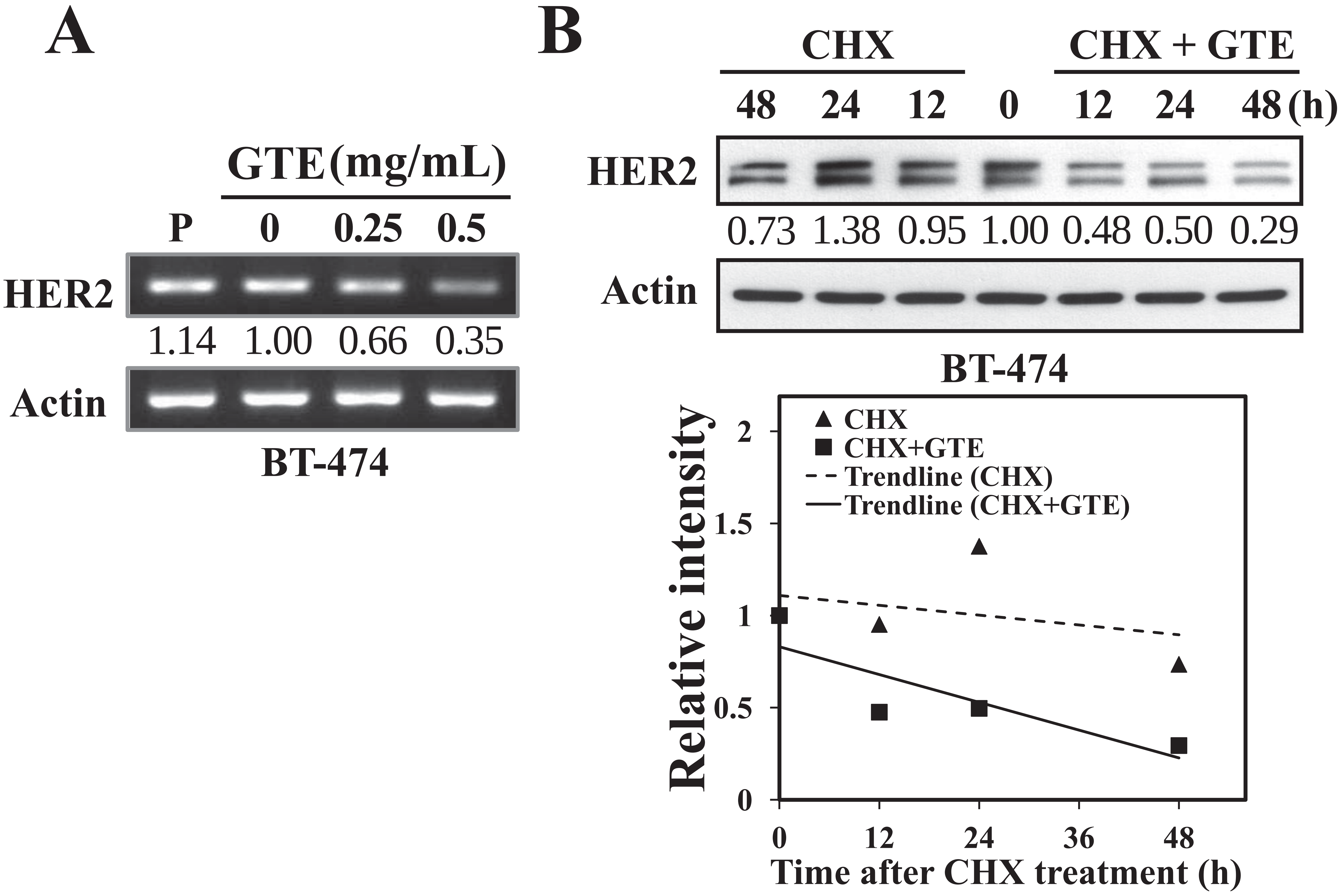

Supplementary Fig. S6: Effect of GTE on the expression of HER2 mRNA and the stability of HER2 protein in BT-474 cells. (A) Cells were treated with or without GTE (0.25 or 0.5 mg/mL) for 24 h. The mRNA level of HER2 was measured by semiquantitative RT-PCR. (B) Cells were pretreated with 20 μg/mL of cycloheximide (CHX) for 30 min and then treated with or without GTE (0.5 mg/mL) for 12, 24, and 48 h. HER2 stability was determined by measuring the protein's half-life. P, parental cells.
